# Cognitive function and knee osteoarthritis: A bidirectional Mendelian randomization study with BMI mediation

**DOI:** 10.1097/MD.0000000000049914

**Published:** 2026-07-24

**Authors:** Yuping Zhang, Qiuxiang Lin, Debiao Yu, Tianxiang Lu, Xiaoting Chen, Yaoyu Lin, Jiawei Qin, Fuchun Wu

**Affiliations:** a Department of Orthopedics, Quanzhou First Hospital Affiliated to Fujian Medical University, Quanzhou, China; b Department of Rehabilitation Medicine, Quanzhou First Hospital Affiliated to Fujian Medical University, Quanzhou, China; c College of Rehabilitation Medicine, Fujian University of Traditional Chinese Medicine, Fuzhou, China; d Provincial Clinical Medicine College of Fujian Medical University, Fuzhou, China; e Department of Rehabilitation Medicine, Fujian Provincial Hospital, Fuzhou, China; f Department of Rehabilitation Medicine, Fuzhou University Affiliated Provincial Hospital, Fuzhou, China.

**Keywords:** body mass index, cognitive function, knee osteoarthritis, Mendelian randomization

## Abstract

This study investigated the causal relationship between cognitive function and knee osteoarthritis (KOA) and whether body mass index (BMI) mediates this effect. A bidirectional Mendelian randomization (MR) analysis was conducted using genome-wide association study summary statistics. The primary analysis used the inverse-variance weighted method, with MR-Egger, weighted median, robust adjusted profile score, and MR-PRESSO applied for sensitivity analyses. Forward MR analyses showed a significant inverse causal effect of cognitive function on KOA risk, with intelligence (OR = 0.843, 95% CI: 0.755–0.941, *P* = .002) and cognitive performance (OR = 0.850, 95% CI: 0.771–0.937, *P* = .001) associated with reduced risk. Reverse MR analyses found no evidence that KOA causally affects cognitive function (Intelligence: β = −0.020, 95% CI:−0.048 to 0.008, *P* = .167; Cognitive performance: β = −0.006, 95% CI:−0.032–0.020, *P* = .661). Mediation analysis showed that BMI partially mediated the effect of intelligence on KOA (indirect β = −0.047, 95% CI:−0.110 to−0.006, *P* = .023), accounting for 27.29% of the total effect. In conclusion, these findings support a unidirectional causal effect of cognitive function on KOA, partially mediated by BMI, highlighting the relevance of cognitive function and weight management in high-risk individuals.

## 1. Introduction

Knee osteoarthritis (KOA) is a complex, multifactorial joint disorder characterized by progressive cartilage degeneration, subchondral bone remodeling, synovial inflammation, and pain sensitization, ultimately leading to impaired physical function and reduced quality of life.^[1]^Established risk factors include advancing age, female sex, a history of knee injury, and excessive joint loading; in particular, overweight (defined as a body mass index (BMI) >25 kg/m^2^) and obesity (defined as a BMI >30 kg/m^2^) are recognized as major contributors to the development of osteoarthritis.^[2,3]^ In addition to increasing mechanical joint loading, overweight and obesity may also contribute to the development of osteoarthritis through metabolic effects and chronic low-grade inflammation.^[4]^ Nevertheless, BMI is not merely a proximal exposure; it is also shaped by upstream determinants such as education, cognitive ability, occupation and income, complicating causal interpretation in conventional epidemiology.^[5,6]^

Cognitive function is increasingly recognized as a determinant of health trajectories across the life course.^[7]^ Previous studies have reported that mild cognitive impairment may increase the incidence of radiographic KOA.^[8]^ Moreover, cognitive function may be associated with dietary patterns and other health-related behaviors, thereby influencing weight control and subsequently affecting joint loading.^[9,10]^ Therefore, cognitive function may indirectly influence the risk of KOA by modulating susceptibility to overweight and obesity. However, in the context of osteoarthritis, observational studies have reported associations between greater pain severity, functional impairment, and poorer cognitive outcomes, yet they offer limited insight into the directionality and underlying mechanisms of these relationships.^[11–13]^ To further elucidate the potential causal relationship between cognitive function and the risk of KOA, as well as to assess the mediating role of BMI, a study design that minimizes confounding is required.

Mendelian randomization (MR) is a commonly used epidemiological approach for causal inference. It leverages genetic variants as instrumental variables to investigate the causal effect of observed cognitive function on KOA.^[14]^ Because genetic variants are randomly allocated at conception, they are not influenced by individual behaviors, social or environmental factors, or other confounders, thereby minimizing the impact of confounding and reverse causation.^[15,16]^ This approach strengthens causal inference compared with conventional observational designs.

In this study, we used MR with instrumental variables derived from genome-wide association study (GWAS) data to examine the causal relationship between cognitive function and KOA. We further performed a two-step analysis to evaluate whether BMI mediates this association. These findings provide additional evidence for the role of cognitive function in KOA risk and inform preventive strategies for high-risk populations.

## 2. Methods

### 2.1. Study design

**Figure 1.**
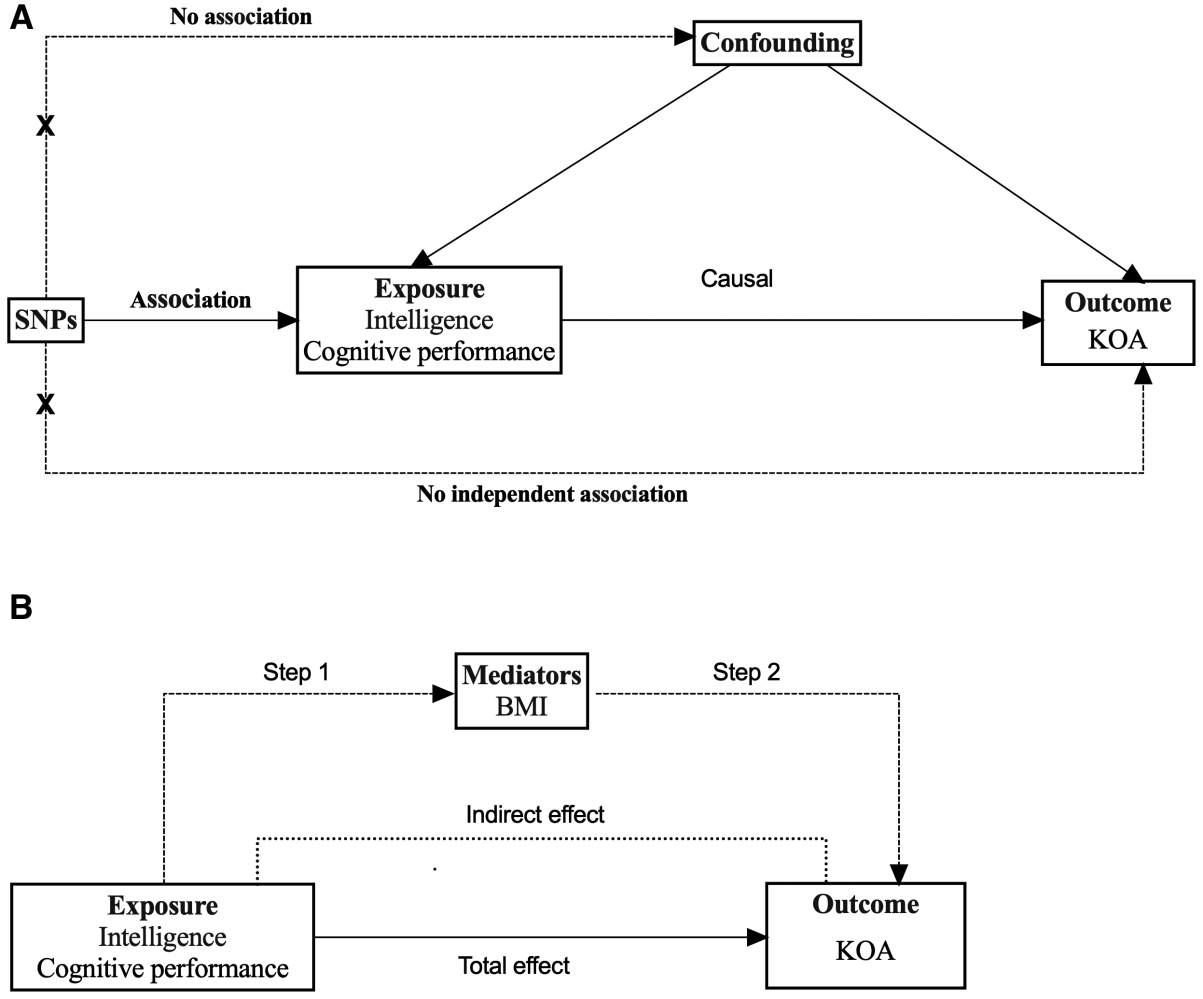
Flowchart of the MR Analysis. BMI = body mass index, KOA = knee osteoarthritis, MR = Mendelian randomization, SNPs = single nucleotide polymorphisms.

We conducted a bidirectional two-sample MR analysis using the largest publicly available GWAS datasets to assess the causal relationship between cognitive function and KOA. In addition, a two-step MR analysis was performed with BMI as a mediator to evaluate its potential mediating role in the association between cognitive function and KOA. To ensure valid causal inference, the instrumental variables (IVs) were required to satisfy three core assumptions: the IVs must be strongly associated with the exposure; the IVs must be independent of any confounders related to the exposure-outcome relationship; and the IVs must influence the outcome solely through the exposure and not via alternative pathways.^[15,17]^ This study followed the latest STROBE-MR reporting guidelines.^[18]^ All statistical analyses were conducted between January 2025 and October 2025 at Quanzhou First Hospital affiliated to Fujian Medical University and collaborating institutions. The study flowchart is presented in Figure 1.

### 2.2. Data sources

This study utilized publicly available GWAS data obtained from the IEU Open GWAS database (https://gwas.mrcieu.ac.uk/) and the FinnGen database (Release R12, https://www.finngen.fi/en). Cognitive function was proxied by intelligence and cognitive performance. The genetic associations for intelligence were derived from a GWAS meta-analysis encompassing 14 cohorts, including both novel and previously reported data, with a total sample size of 269,867 individuals.^[19]^ Intelligence scores were derived from a range of neurocognitive tests primarily assessing fluid cognitive ability, with higher scores indicating better cognitive function. Genetic associations for cognitive performance were obtained from a meta-analysis dataset combining data from the UK Biobank (UKB) and the Cognitive Genomics Consortium (COGENT), comprising a total sample size of 257,841 individuals.^[20]^ Cognitive performance scores were based on participants’ results in a verbal-numerical reasoning test, with higher scores reflecting better cognitive function. Within the FinnGen database, KOA was represented by the phenotype “Gonarthrosis,” encompassing 61,356 cases and 315,115 controls.^[21]^ In the FinnGen database, KOA diagnoses were defined based on national hospital discharge records and death registry data. Individuals with the International Classification of Diseases, 10th Revision (ICD-10) code M17 recorded in the registry were classified as KOA cases. Genetic associations for BMI were obtained from the Genetic Investigation of Anthropometric Traits Consortium dataset, comprising a total sample size of 339,224.^[22]^ BMI was calculated as weight divided by height squared. All samples were of European ancestry, with no overlap identified between cohorts. Data sources and sample sizes used in this study are summarized in Table 1.

**Table 1:** The GWAS data source details.

Phenotype	Data source	Consortium	Sample size (case/control)	Ancestry	PMID
Intelligence	IEU openGWAS	14 cohorts	269,867	European	29,942,086
Cognitive performance	IEU openGWAS	UKB and COGENT	257,841	European	30,038,396
Gonarthrosis	FinnGen	NA	376,417 (61,356/315,115)	European	36,653,562
Overweight	IEU open GWAS	GIANT	339,224	European	25,673,413

COGENT = Cognitive Genomics Consortium, GIANT = Genetic Investigation of Anthropometric Traits, IEU OpenGWAS = Integrative Epidemiology Unit Open Genome-Wide Association Studies database, NA = not applicable, UKB = United Kingdom Biobank.

### 2.3. Ethics statement

This study was based exclusively on publicly available, de-identified GWAS summary statistics. No individual-level data were collected, and no human participants were directly involved. All original studies contributing to the GWAS datasets received ethical approval from their respective institutional review boards, and informed consent was obtained from participants. Therefore, no additional ethical approval was required for this secondary data analysis. The study was conducted in accordance with the principles of the Declaration of Helsinki.

### 2.4. Selection of genetic instrumental variables

Single nucleotide polymorphisms (SNPs) significantly associated with the exposure (*P* < 5 × 10^−8^) were selected from GWAS as initial instrumental variables. Then, we employed a reference panel from the 1000 Genomes Project (European population) to exclude SNPs with linkage disequilibrium (LD) (*r*^2^ < 0.001 and clumping window >10,000 kb).^[23]^ Harmonization procedures were subsequently conducted to align the effect alleles between the exposure and outcome datasets. Palindromic SNPs with ambiguous strand orientation or inconsistent allele information were excluded during the harmonization process. To minimize weak instrument bias, only SNPs with an *F*-statistic > 10 were retained as valid instrumental variables.^[24]^ The F-statistic is a measure of the strength of the instrumental variable, calculated as F = R^2^ (N–K–1)/ k(1–R^2^), where R^2^ = 2 × EAF × (1–EAF) × β^2^.^[25,26]^ Finally, the LDtrait tool (https://ldlink.nci.nih.gov/?tab=ldtrait) was used to manually exclude potential confounders.^[27]^ LDtrait was used to identify phenotype associations in linkage disequilibrium with each SNP. SNPs were excluded if they showed genome-wide significant associations with KOA risk factors or potential confounders. The SNPs included in the statistical analysis after exclusion are listed in Supplementary Table S1, Supplemental Digital Content 1.

### 2.5. Statistical analyses

We used the “TwoSampleMR” package in R version 4.4.1 to estimate the causal relationship between cognitive function and KOA. To enhance the robustness of our findings, we applied 5 complementary MR methods: inverse-variance weighted (IVW) analysis, MR-Egger regression, weighted median (WM) estimator, MR-Robust adjusted profile score (MR-RAPS), and MR Pleiotropy Residual Sum and Outlier (MR-PRESSO). IVW served as the primary analytical method, combining the Wald ratio estimates of individual SNPs into an overall causal effect for each risk factor. Each Wald estimate was derived by dividing the SNP-outcome association by the corresponding SNP-exposure association.^[28]^ In the presence of horizontal pleiotropy, MR-Egger provides a more robust estimate of the causal effect.^[29,30]^ The WM method yields accurate and robust causal estimates when at least 50% of the instrumental variables are valid.^[31]^ MR-RAPS is a likelihood-based adjustment method suitable for situations with pleiotropic or weak instrument bias, providing more reliable effect estimates in complex scenarios.^[32]^ The MR-PRESSO method identifies outlier variants that may bias causal estimates and provides corrected estimates after their removal.^[33]^ Heterogeneity across individual SNPs was assessed using Cochran’s Q test.^[34,35]^ A random-effects model was applied when significant heterogeneity was detected (*P* < .05); otherwise, a fixed-effects model was used. Horizontal pleiotropy was assessed by examining the *P*-value of the intercept from MR-Egger regression.^[29]^ In the presence of significant horizontal pleiotropy (*P* < .05), the corresponding results were considered unreliable and excluded from interpretation. Sensitivity analyses were performed through visual inspection of scatter plots, funnel plots, and leave-one-out plots. Finally, reverse MR analyses were conducted, treating KOA as the exposure and cognitive function as the outcome, to evaluate the potential causal effect of KOA on cognitive function.

Mediation analysis was conducted using a two-step MR approach to investigate the mediating role of BMI in the relationship between cognitive function and KOA. First, the overall causal effect of cognitive function on KOA (β_total_) is estimated based on the Two-Sample MR analysis. Subsequently, the mediation effect is verified through a two-step method: in the first step, the causal effect of cognitive function on BMI (β_1_) is estimated, and in the second step, the causal effect of BMI on KOA (β_2_) is estimated. If cognitive function significantly affects BMI, which in turn influences KOA, the mediation effect of BMI will be calculated using the product coefficient method (β_mediation_ = β_1_ × β_2_). The standard error of the mediation effect will be calculated using the δ method, and the proportion of the mediation contribution will be derived using the proportion effect method (Proportion mediated = β_mediation_/ β_total_).^[36,37]^ This mediation framework assumes valid instrumental variables for each causal pathway and no substantial interaction between the exposure and mediator.

Given that two primary forward MR analyses were performed, a Bonferroni correction was applied to control for type I error. The adjusted significance threshold was set at *P* < .025 (0.05/2). Reverse MR and mediation analyses were considered secondary or exploratory analyses and were interpreted with caution.

## 3. Results

### 3.1. Causal relationship between cognitive function and KOA

**Figure 2.**
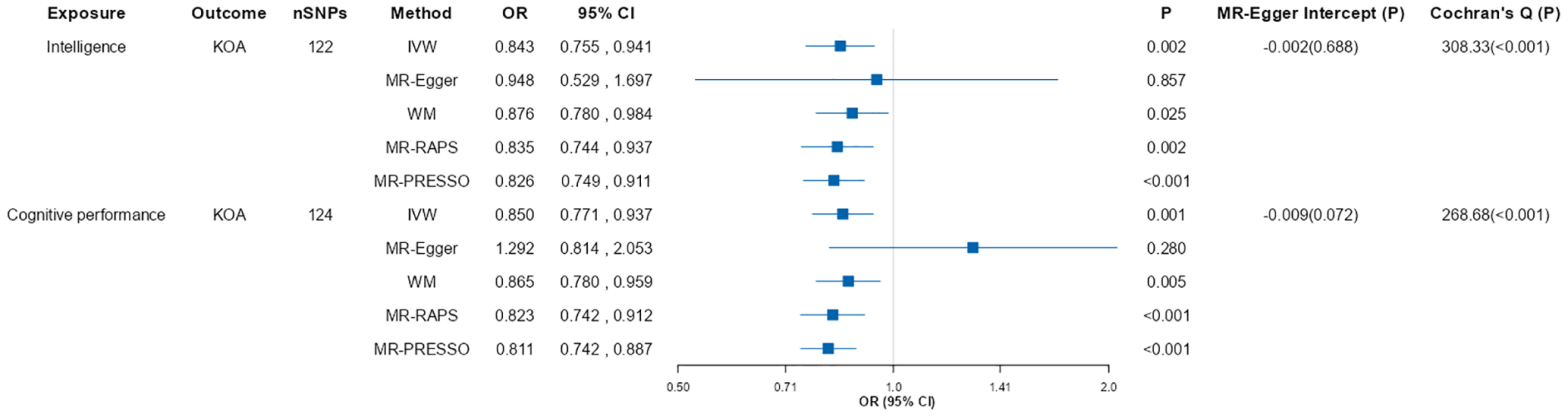
MR analysis of cognitive function and KOA. CI = confidence interval, IVW = inverse-variance weighted, KOA = knee osteoarthritis, MR = Mendelian randomization, OR = odds ratio, WM = weighted median.

**Figure 3.**
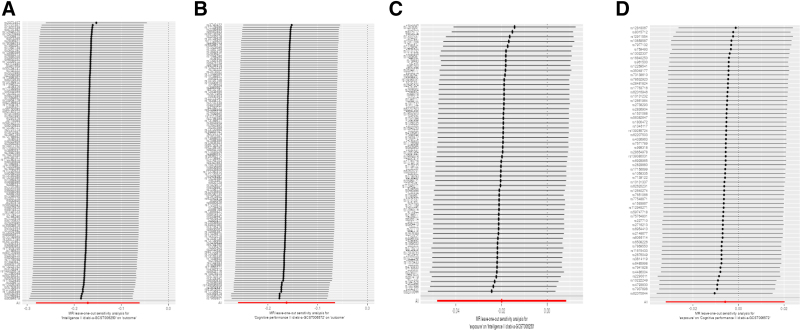
Leave-one-out sensitivity analyses. (A) Intelligence on KOA, (B) cognitive performance on KOA, (C) reverse MR analysis of KOA on Intelligence, (D) reverse MR analysis of KOA on cognitive performance. KOA = knee osteoarthritis, MR = Mendelian randomization.

**Figure 4.**
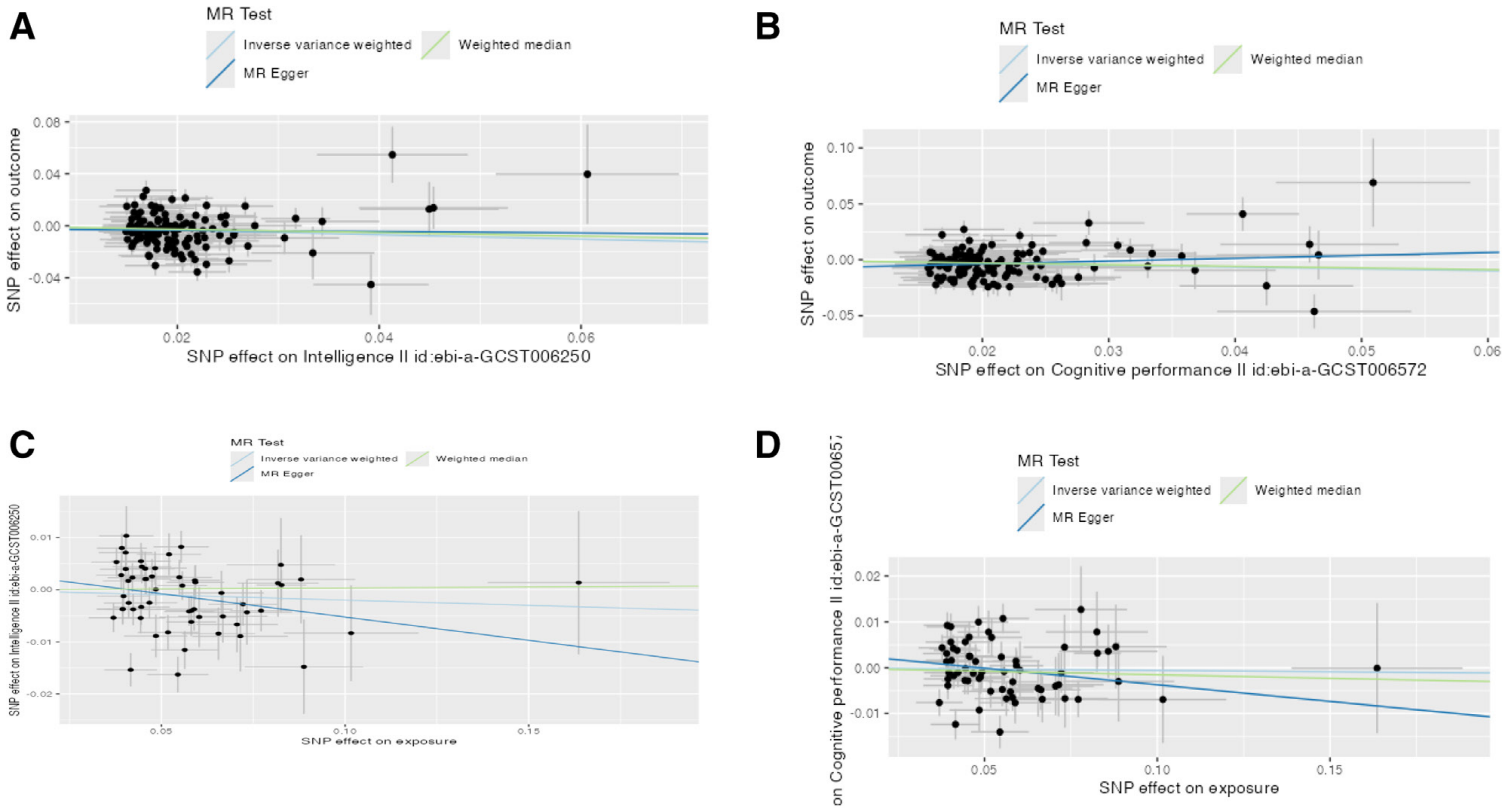
Scatter plots of genetic instrument-outcome associations. (A) Intelligence on KOA, (B) cognitive performance on KOA, (C) reverse MR analysis of KOA on Intelligence, (D) reverse MR analysis of KOA on cognitive performance. KOA = knee osteoarthritis, MR = Mendelian randomization.

Significant heterogeneity was observed in the forward MR analyses (*P* < .001); therefore, a random-effects IVW model was applied. The IVW analysis demonstrated significant inverse associations (intelligence: OR = 0.843, 95% CI: 0.755–0.941, *P* = .002; cognitive performance: OR = 0.850, 95% CI: 0.771–0.937, *P* = .001). Findings from the WM, MR-RAPS, and MR-PRESSO methods were consistent with the IVW estimates. After Bonferroni correction, both associations remained statistically significant across the two primary analyses (adjusted significance threshold, *P* < .025). The MR-Egger intercept test did not detect evidence of horizontal pleiotropy (intelligence: *P* = .688; cognitive performance: *P* = .072), suggesting that the associations were unlikely to be driven by pleiotropic effects (see Figure 2). Furthermore, leave-one-out analyses and scatter plots further supported the robustness of the findings (see Figures 3A–B and 4A–B). Funnel plots also showed no obvious evidence of asymmetry (see Supplementary Figure S1a-b, Supplemental Digital Content 2).

### 3.2. Causal relationship between KOA and cognitive function

**Figure 5.**
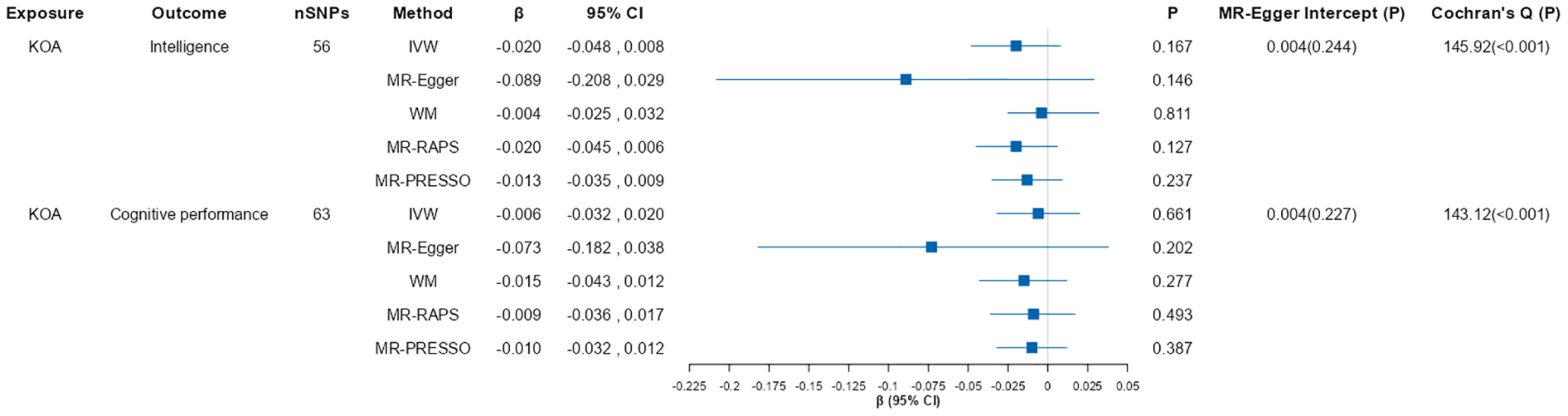
MR analysis of KOA and cognitive function. CI = confidence interval, KOA = knee osteoarthritis, MR = Mendelian randomization, WM = weighted median.

Significant heterogeneity was observed in the reverse MR analyses (*P* < .001); therefore, a random-effects IVW model was applied. The IVW estimates did not indicate significant causal effects (intelligence: β = −0.020, 95% CI:−0.048–0.008, *P* = .167; cognitive performance: β = −0.006, 95% CI:−0.032–0.020, *P* = .661). Results from MR-Egger, the WM, MR-RAPS, and MR-PRESSO methods were consistent with these findings. The MR-Egger intercept test did not detect evidence of horizontal pleiotropy (intelligence: *P* = .244; cognitive performance: *P* = .227), suggesting that the estimates were unlikely to be affected by pleiotropic effects (see Figure 5). Furthermore, leave-one-out analyses and scatter plots further supported the robustness of the findings (see Figures 3C–D and 4C–D). Funnel plots also showed no obvious evidence of asymmetry (see Supplementary Figure S1c-d, Supplemental Digital Content 2).

### 3.3. Mediation analysis

**Figure 6.**
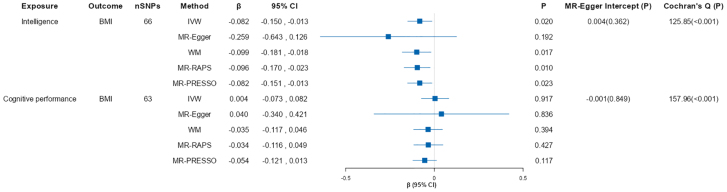
MR analysis of cognitive function on BMI. BMI = body mass index, CI = confidence interval, MR = Mendelian randomization, IVW = inverse-variance weighted, WM = weighted Median.

**Figure 7.**
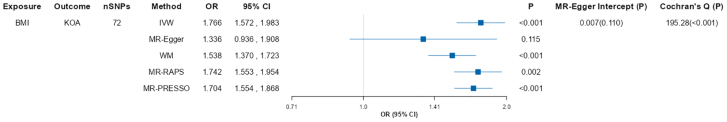
MR analysis of BMI on KOA. BMI = body mass index, KOA = knee osteoarthritis, IVW = inverse-variance weighted, MR = Mendelian randomization, WM = weighted Median

**Figure 8.**
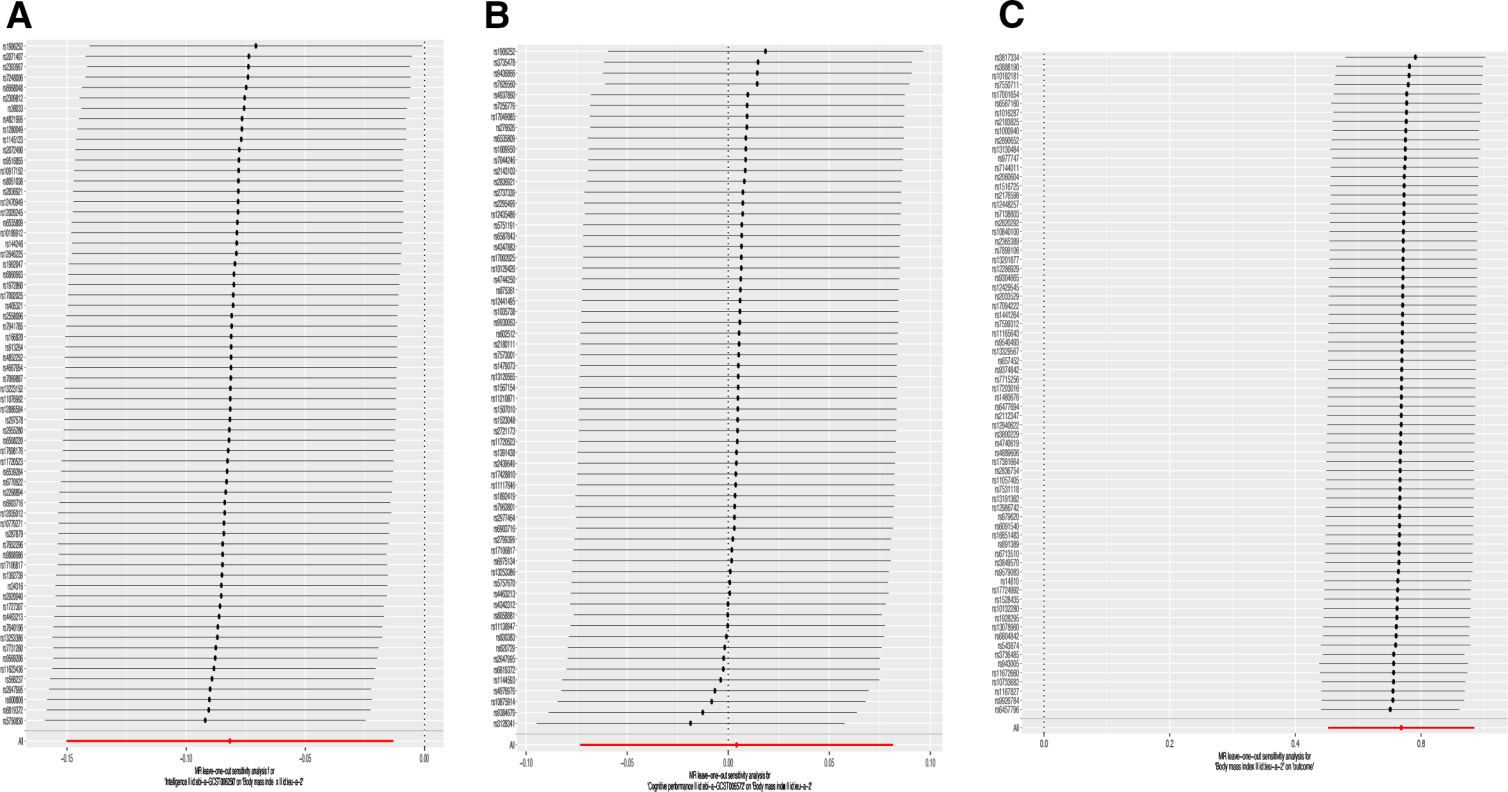
Leave-one-out sensitivity analyses. (A) Intelligence on BMI, (B) cognitive performance on BMI, (C) BMI on KOA. BMI = body mass index, KOA = knee osteoarthritis.

**Figure 9.**
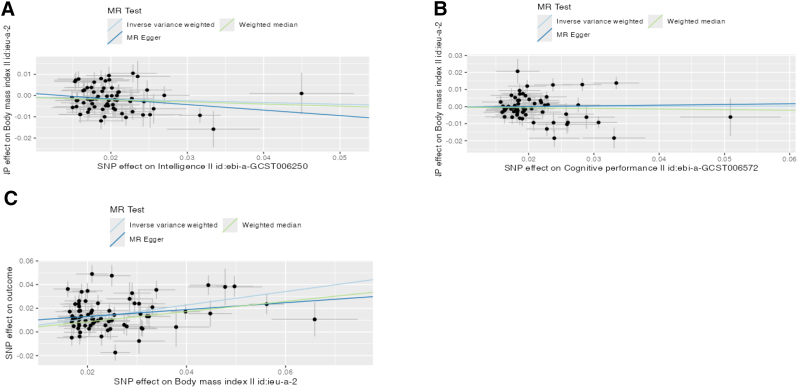
Scatter plots of genetic instrument-outcome associations. (A) Intelligence on BMI, (B) cognitive performance on BMI, (C) BMI on KOA. BMI = body mass index, KOA = knee osteoarthritis, MR = Mendelian randomization, SNPs = single nucleotide polymorphisms.

In the MR analyses examining the relationships between cognitive function and BMI, and between BMI and KOA, significant heterogeneity was observed in all comparisons (*P* < .001); therefore, random-effects IVW models were applied. The results indicated a significant negative causal effect of intelligence on BMI (β = −0.082, 95% CI:−0.150 to −0.013, *P* = .020), whereas no causal association was observed between cognitive performance and BMI (β = 0.004, 95% CI:−0.073–0.082, *P* = .917). In the analysis of BMI and KOA, a positive causal association was observed (OR = 1.766, 95% CI: 1.572–1.983, *P* < .001). MR-Egger intercept tests did not detect evidence of horizontal pleiotropy (intelligence: *P* = .362; cognitive performance: *P* = .849; BMI: *P* = .110), indicating that the estimates were unlikely to be affected by pleiotropic effects (see Figures 6–7). Moreover, leave-one-out analyses and scatter plots further supported the robustness of these estimates (see Figures 8–9). Funnel plots also showed no obvious evidence of asymmetry (see Supplementary Figure S1e-g, Supplemental Digital Content 2).

#### 3.3.1. Mediation effect

The causal effect of intelligence on KOA (β_total = −0.171, 95% CI:−0.281–−0.061), the effect of intelligence on BMI (β_1_ = −0.082, 95% CI:−0.150 to −0.013), and the effect of BMI on KOA (β_2_ = 0.569, 95% CI: 0.452–0.685) were obtained from previous MR analyses. Mediation analysis incorporating these estimates revealed a significant indirect effect of BMI in the causal pathway from intelligence to KOA (β = −0.047, 95% CI:−0.110–−0.006, *P* = .023), accounting for 27.29% of the total effect. This indirect effect remained below the Bonferroni-adjusted significance threshold; however, given the exploratory nature of mediation MR, the finding should be interpreted with caution. Detailed results are provided in Table 2.

**Table 2:** Mediation Effect of BMI.

Mediation	β_total_ (95% CI)	β_1_ (95% CI)	β_2_ (95% CI)	β_mediation_ (95% CI)	*P*	Proportion mediated
BMI	−0.171 (−0.281, −0.061)	−0.082 (−0.150, −0.013)	0.569 (0.452, 0.685)	−0.047 (−0.110, −0.006)	0.023	27.29%

**β**_**total**_, **β**_**1**_, and **β**_**2**_ were derived from the ORs obtained using IVW and converted to β values using the formula **β = ln (OR**).

A *P*-value of <.05 was considered statistically significant.

BMI = body mass index, CI = confidence interval, OR = odds ratio, IVW = inverse-variance weighted method.

## 4. Discussion

This study presents a comprehensive bidirectional MR analysis to investigate the causal relationship between cognitive function and KOA. The primary findings indicate that higher cognitive function (intelligence and cognitive performance) is significantly associated with a reduced risk of KOA. Mediation analysis further showed that BMI mediates approximately 27.29% of the total causal effect of intelligence on KOA. In contrast, no evidence supported a reverse causal effect of KOA on cognitive function. These findings provide novel genetic evidence regarding upstream determinants of joint health and highlight the multifaceted role of cognitive function in KOA disease progression.

The inverse association between cognitive function and KOA reinforces the view that cognitive ability is an important driver of long-term health. Mediation analysis showed that BMI accounted for 27.29% of the total effect, suggesting that individuals with higher intelligence may be better able to maintain a healthy body weight. Individuals with higher cognitive function may reduce the risk of obesity by adopting healthier dietary habits, maintaining regular physical activity, and demonstrating greater awareness of health-related risks.^[38]^ Notably, approximately 73% of the protective effect of intelligence remained independent of BMI. This finding suggests the presence of additional biological or behavioral pathways, including systemic inflammation, altered pain processing, and socioeconomic pathways related to educational attainment and occupational exposures.^[39–42]^ While intelligence demonstrated a significant negative causal effect on BMI, no such association was observed for cognitive performance (*P* = .917). This may be because the cognitive performance measure used in this study was based on scores from a verbal-numerical reasoning test. Individuals with higher verbal-numerical reasoning scores often attain higher levels of education, which may increase the likelihood of entering occupations that require lower physical demands. This, in turn, may reduce mechanical loading on the knee joint and subsequently lower the risk of developing KOA.^[43,44]^

Regarding directionality, the reverse MR analysis clarified the relationship between KOA and cognitive decline. Although several observational studies have suggested that chronic pain and functional limitations in patients with KOA may lead to declines in cognitive function.^[12,45]^ However, a recent combined NHANES and MR study reported no significant causal relationship between genetically predicted osteoarthritis and the risk of all-cause dementia.^[46]^ While that study suggested depression as a potential mediator between osteoarthritis and cognitive outcomes, our study further highlights BMI as an important metabolic mediator in the forward causal pathway. Collectively, these findings suggest that previously reported clinical associations may be attributable to shared environmental factors or psychological intermediaries rather than a direct neurobiological consequence of joint degeneration.

Strikingly, significant heterogeneity was observed across the forward, reverse, and mediation MR analyses (*P* < .001). Such heterogeneity is expected, as KOA is a complex, multifactorial joint disorder involving diverse pathological processes, including cartilage degeneration and synovial inflammation.^[47]^ Furthermore, the genetic proxies for cognitive function were derived from diverse neurocognitive tests, ranging from fluid cognitive ability to verbal-numerical reasoning, which may introduce biological variability. To account for this, we utilized random-effects IVW models to provide more conservative and robust causal estimates.^[48]^ The consistency of our findings across complementary methods (such as MR-PRESSO, which corrected for potential outlier variants) further reinforces that the observed heterogeneity does not undermine the fundamental causal protection identified in this study.

Clinically, these findings have several important implications. First, cognitive function should be acknowledged as a distal risk factor for KOA. Second, the substantial mediation by BMI highlights that weight management represents a critical intervention target, particularly for individuals with lower intelligence scores. Third, for individuals whose cognitive profiles indicate a risk independent of BMI, interventions should focus on ergonomic education and minimizing occupational joint loading.

Despite the strengths of this study, including the use of large-scale GWAS datasets and robust sensitivity analyses, several limitations should be acknowledged. First, all participants were of European ancestry, limiting the generalizability of the findings. Second, although we applied the MR-Egger intercept test and queried LDtrait to reduce horizontal pleiotropy, residual pleiotropic effects cannot be completely excluded. Third, KOA was defined using the FinnGen “gonarthrosis” phenotype, which may include heterogeneous disease states; therefore, our estimates reflect genetic liability to registry-defined KOA rather than a clinically uniform subtype. Finally, the two-step MR mediation analysis assumes valid instrumental variables and no substantial interaction between the exposure and the mediator. Although BMI was selected as a biologically plausible mediator, other potential mediators, such as physical activity and systemic inflammation, were not evaluated and should be explored in future studies.

## 5. Conclusion

In this study, we performed MR analyses using large-scale GWAS data on cognitive function and KOA. Our findings indicate a unidirectional causal relationship, whereby higher cognitive function is associated with a reduced risk of KOA. BMI partially mediates the association between intelligence and KOA. Future studies should confirm these findings in diverse populations and explore mechanisms beyond BMI.

## Acknowledgments

We thank all consortia and studies for publicly sharing GWAS data.

## Author contributions

**Formal analysis:** Yuping Zhang.

**Investigation:** Yuping Zhang.

**Conceptualization:** Qiuxiang Lin.

**Data curation:** Qiuxiang Lin, Xiaoting Chen.

**Methodology:** Qiuxiang Lin, Debiao Yu.

**Software:** Debiao Yu.

**Supervision:** Tianxiang Lu.

**Validation:** Tianxiang Lu.

**Visualization:** Tianxiang Lu.

**Project administration:** Yaoyu Lin.

**Writing – original draft:** Yuping Zhang.

**Writing – review & editing:** Jiawei Qin, Fuchun Wu.




