# Supplementary material for: Cognitive function and knee osteoarthritis: A bidirectional Mendelian randomization study with BMI mediation
Source: Medicine (Baltimore). 2026 Jul 24;105(30):e49914. doi: 10.1097/MD.0000000000049914 (PMC13406376; doi:10.1097/MD.0000000000049914)
Supplement: Supplementary file 2 [file medi-105-e49914-s002.pdf]

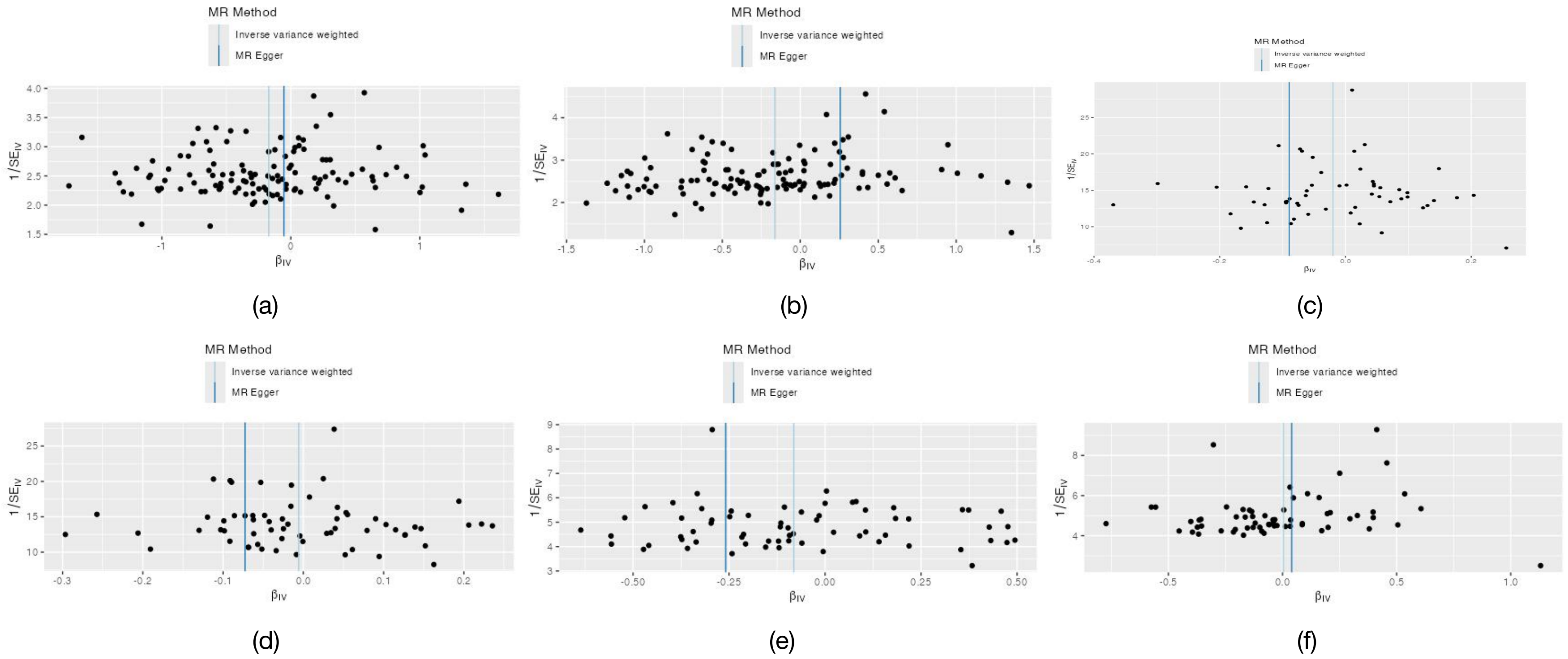

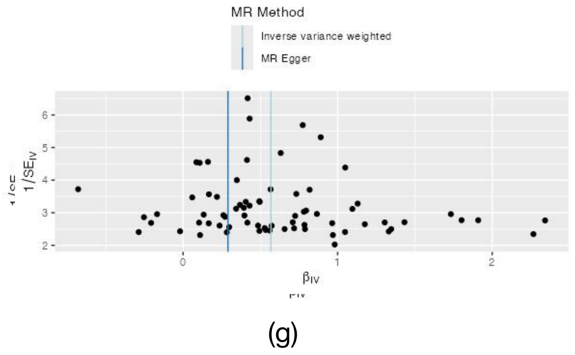

Appendix Figure S1, Funnel plot

(a) Intelligence on KOA;

(b) Cognitive performance on KOA;

(c) reverse MR analysis of KOA on Intelligence;

(d) reverse MR analysis of KOA on Cognitive performance;

(e) Intelligence on BMI;

(f) Cognitive performance on BMI;

(g) BMI on KOA
